# Transcriptional Alteration of Gene Biomarkers in Hemocytes of Wild *Ostrea edulis* with Molecular Evidence of Infections with *Bonamia* spp. and/or *Marteilia refringens* Parasites

**DOI:** 10.3390/pathogens9050323

**Published:** 2020-04-26

**Authors:** Paolo Cocci, Alessandra Roncarati, Martina Capriotti, Gilberto Mosconi, Francesco Alessandro Palermo

**Affiliations:** School of Biosciences and Veterinary Medicine, University of Camerino, Via Gentile III Da Varano, I-62032 Camerino (MC), Italy; paolo.cocci@unicam.it (P.C.); alessandra.roncarati@unicam.it (A.R.); martina.capriotti@unicam.it (M.C.); gilberto.mosconi@unicam.it (G.M.)

**Keywords:** flat oyster, molecular analysis, *Bonamia ostreae*, *Marteilia refringens*, hemocyte, biomarkers, qRT-PCR, gene expression

## Abstract

The European flat *Ostrea edulis* is highly susceptible to intracellular parasitic infections, particularly bonamiosis and marteiliosis. The defensive response of oyster to both bonamiosis and marteiliosis is typically mediated by hemocytes, which play a pivotal role in immune system homeostasis. In the present study, we first used a DNA-based tool in order to rapidly and specifically detect the presence of parasites in oysters from natural banks in the middle Adriatic Sea. In a second step, we used qRT-PCR to analyze the mRNA levels of a set of genes (i.e., superoxide dismutase (SOD), glutathione S-transferase (GST), metallothionein (MT), heat shock protein (HSP) 70 and 90, inhibitor of apoptosis (IAP), fas ligand (FAS), galectin (GAL) and extracellular superoxide dismutase (Ec-SOD)) expressed by hemocytes of flat oysters infected by the parasites, present singularly or in combination, compared to hemocytes from non-infected specimens. The results indicate that the presence of parasite DNA may be associated to a general upregulation of host genes related to apoptosis, detoxification and oxidative stress protection, with the exception of Ec-SOD, whose trend to a downregulation might reflect a mechanism for parasite escape before internalization.

## 1. Introduction

*Ostrea edulis* is the native European oyster species, with a natural range that typically includes all the Mediterranean basin. In Europe, natural beds of *O. edulis* are present in several regions, including the west coast of Ireland, the Limfjord region of Denmark and the coasts of the Adriatic Sea. The northern and middle Adriatic Sea represents an important site where natural beds subsist, as reported in Powered (2014) [1] and by Petochi et al. [2]. In the recent years, wild populations of *O. edulis* have shown a decline throughout European waters most likely due to multiple factors, including combined habitat loss and degradation [3] and pathogenic diseases [4,5,6]. Most of these infectious diseases have now been found to be the result of intracellular infections by protozoan parasites such as, among others, *Bonamia ostreae* and *Marteilia refringens*. 

*Bonamia ostreae* is the causative agent of bonamiosis, a disease of oyster hemocytes, which has caused extensive mortalities among *O. edulis* leading to a decline in distribution range for this species [7]. The parasite causes severe hemocyte destruction and heavy hemocytic infiltration in the connective tissue of target organs such as gills, mantle, digestive gland and gonads [8]. Similarly, *M. refringens* is a protozoan of great concern to oysters, including *O. edulis*, as this parasite is responsible for the marteiliosis disease that causes mass mortalities in this species. Infection with *M. refringens* may interfere with oyster feeding, absorption of organic matter and glycogen storage, reducing the condition index (i.e., CI = dry tissue weight/dry shell weight × 100; a measure of the ecophysiological state of an individual oyster) [9,10,11,12]. In the Adriatic Sea, data on the presence/absence of these parasites (including cases of co-infection) in flat oysters are quite scant. The last known records of *M. refringens* and *Bonamia* spp. in natural banks of European flat oysters along the Italian coasts of the central-southern Adriatic Sea are from 2004 and 2010, respectively [6,13].

Hemocytes play a pivotal role in immune system homeostasis, executing several immune functions, including defense, which remains the main cellular immune response of bivalve mollusks to a pathogen [14]. Once the pathogen has been phagocytosed, the hemocyte will activate defense mechanisms that produce reactive oxygen intermediates to kill the pathogen [15]. However, there is evidence that various pathogens, including *B. ostreae*, survive inside of phagocytes, also escaping post-phagocytosis mechanisms [16,17,18]. The majority of studies on the interactions between hemocytes and parasites have been carried out by using cellular approaches [14,17,19,20]. Only a few works have examined the molecular responses of *O. edulis* oysters against parasites with a focus on, among others, the modulation of expression of genes implicated in detoxification, oxidative stress protection and immune response, such as glutathione S-transferase omega (OGST), superoxide dismutase (SOD) and galectin (GAL), or in the apoptosis pathway, such as inhibitor of apoptosis (IAP) and fas ligand (FAS) [21,22,23].

In this context, the aim of our study was to investigate some of the molecular mechanisms involved in mediating *O. edulis* oxidative stress defense against *Bonamia* spp. and *M. refringens.* As a first step, we used a PCR detection in order to rapidly and specifically detect the presence of parasites in oysters from natural banks in the middle Adriatic Sea (Marche Region, Central Italy). Oysters are of particular interest to the Marche Region because of their ecological and economic importance, especially as diversification in farmed molluscan species [24]. In a second step, we used qRT-PCR to analyze the mRNA levels of a set of genes (i.e., SOD, extracellular SOD (Ec-SOD), GST, metallothionein (MT), heat shock protein (HSP) 70 and 90, IAP, FAS and GAL) differentially expressed by hemocytes of flat oysters infected by the parasites, present singularly or in combination, compared to hemocytes from non-infected specimens.

## 2. Results

### 2.1. PCR Detection of B. ostreae, B. exitiosa and M. refringens

When PCR was performed using gills, an overall *Bonamia* infection of 35.4% was observed, with 14/48 oysters (29.2%) infected only by *B. ostreae* and 3/48 (6.2%) having concurrent infection of *B. exitiosa* and *B. ostreae.* No samples were found to be infected only by *B. exitiosa.* Interestingly, molecular detection of the parasite carried out using digestive gland confirmed only 10/48 (20.8%) of the oysters to harbor *Bonamia* parasite DNA. 

*M. refringens* DNA in digestive gland, detected by nested PCR, was found in 14/48 different organisms, i.e., 29.2% of the total number of *O. edulis* analyzed. When PCR was performed using gills, amplicons of 201 bp were confirmed for 10 samples (20.8%).

Overall, co-occurrence of the two parasites was observed in both gill (8/48, 16.7%) and digestive gland (7/48, 14.86%).

### 2.2. Gene Expression Analysis 

**Figure 1:**
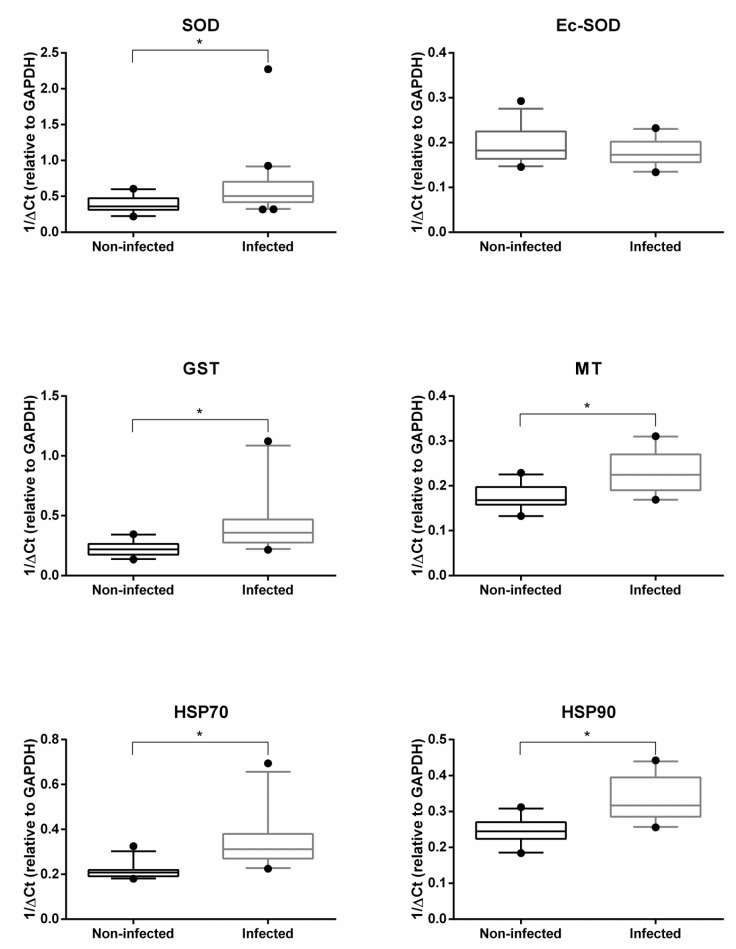
Box-and-whisker plots of superoxide dismutase (SOD), extracellular superoxide dismutase (Ec-SOD), glutathione S-transferase (GST), metallothionein (MT), heat shock protein (HSP)70 and HSP90 transcription values (medians and 95% confidence intervals) in hemocytes of infected and non-infected groups of oysters *Ostrea edulis*. Data are shown as 1/ΔCT (relative to glyceraldehyde 3-phosphate dehydrogenase (GAPDH)) value, and statistical significance (*p < 0.01) was evaluated by Student’s *t*-test.

**Figure 2:**
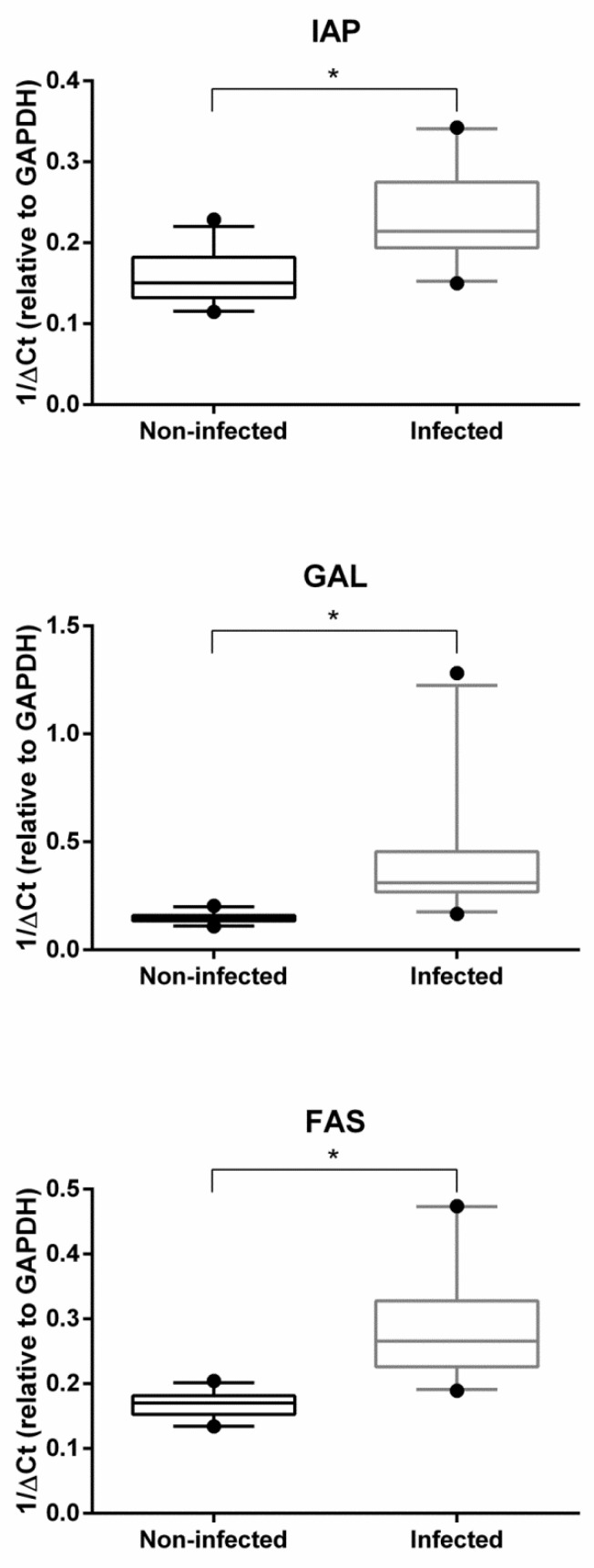
Box-and-whisker plots of inhibitor of apoptosis (IAP), fas ligand (FAS) and galectin (GAL) transcription values (medians and 95% confidence intervals) in hemocytes of infected and non-infected groups of *Ostrea edulis* oysters. Data are shown as 1/ΔCT (relative to glyceraldehyde 3-phosphate dehydrogenase (GAPDH)), and statistical significance (*p < 0.01) was evaluated by Student’s *t*-test.

Figure 1 and Figure 2 show the hemocyte expression levels of target genes involved in detoxification and oxidative stress responses (i.e., SOD, Ec-SOD, GST, MT, HSP70 and HSP90) or in immune response and apoptosis pathways (i.e., IAP, FAS and GAL) between infected and non-infected groups of *O. edulis*. With the exception of Ec-SOD, which was found to be slightly downregulated by the presence of parasites, all other genes revealed significantly higher expression values in infected oysters then in non-infected ones. 

### 2.3. Principal Component Analyses Results

**Figure 3:**
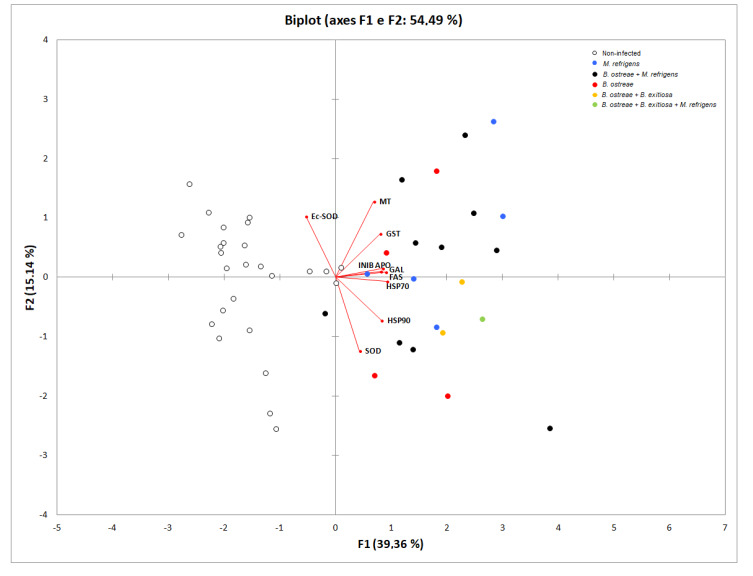
Biplots illustrating the main results of PCA analyses.

PCA was used to evaluate the potential link(s) between the gene biomarkers and parasitic infections and explained approximately 54.49% of the total variance (PC1, 39.36%; PC2, 15.14%) (Figure 3). The biplot shows that non-infected oysters, without presence of parasite DNA, are mostly grouped on the left part of the biplot, and most of the infected specimens are predominantly spread in the right part of the biplot. Data also confirm the positive correlation among genes involved in apoptosis and immune responses.

## 3. Discussion

The wide availability of molecular methods allows sensitive and specific diagnosis of parasite infection in mollusks, including oysters. In this context, several PCR amplification methods have been developed for the rapid detection and discrimination of intracellular parasitic infections such as *Bonamia* spp. and *M. refringens*, both responsible for major mortalities among *O. edulis* [5,7,25]. For the purpose of this study, we used two different procedures of amplification by PCR in order to carry out a rapid screening test to be used for indicating the presence or absence of the parasites in oyster target tissues (i.e., gill for *B. ostreae* and digestive gland for *M. refringens*). Both methods have been proved to be efficient diagnostic tools showing strong analytical specificity and sensitivity. In the latter respect, results also demonstrate the importance of using the key target tissues for early diagnosis. However, one interesting finding we found is that, in some oysters, *Marteilia refringens* parasites were present in gill epithelia but not in digestive glands. This parasite localization is considered unusual [26], even if few previous studies reported the presence of young plasmodial stages of *M. refringens* in gill tissues of *O. edulis* [27,28]. Taken together, these findings suggest that the real life cycle of *M. refringens* is not yet entirely known. In addition, the gill epithelium may be considered as a potential route of entry for *M. refringens*, which has been demonstrated to penetrate its host through different epithelia, including gills, and move through systemic circulation [26,29]. For this reason, molecular detection of oyster parasitic pathogens can be very useful to improve the understanding of the parasite cycle within the host and to observe early infections which are not easy to detect by routine histological observation [30]. The results of our study support previous findings showing the presence of *B. ostreae, B. exitiosa* and *M. refringens* in natural banks of the flat oyster (*Ostrea edulis*) along the Italian coasts of the Adriatic Sea [6,13]. In addition, the work by Narcisi et al. [6] was the first to use molecular analysis for confirming the presence of *B. ostreae* in the Manfredonia Gulf since 1990. Interestingly, the molecular diagnosis has also been used to detect cases of co-infection of *M. refringens* and *Bonamia spp.* in *Ostrea edulis* cultured along the Spanish Mediterranean coast [26].

Following detection of parasite DNA, a molecular approach to the investigation of *O. edulis* responses to infection was used in our study. In fact, expression levels of specific genes associated with *O. edulis* cell stress defense, communication and immune response against parasites were investigated in hemocytes, with the aim of detecting changes in molecular profiles. The comparison of these gene mRNA levels between infected and non-infected oysters provided interesting information for the interpretation of the response to parasitic infections. For example, the increased expression of GST and MT in the presence of parasite DNA might be related to an increase of cytotoxic components generated during an immune response, as demonstrated in other mollusks [31]. In fact, GST plays a critical role in cell detoxification of toxic compounds during an immune response [32,33]. Similarly, MTs, which are known as small heavy-metal-binding proteins, protect cells from exposure to oxidants and electrophiles [34]. Previous reports indicated that *B. ostreae* challenged hemocytes displayed a significant reduction of reactive oxygen species (ROS) production due to an activation of genes involved in cellular detoxification [14,22]. Desclaux-Marchand et al. [35] reported an upregulation of MT gene in the cockle (*Cerastoderma edule*) under parasitism. The authors also showed significant effects of both Cd and parasitism on MT gene induction, thus re-emphasizing the long-standing controversial question about the use of MT as a biomarker of metal contamination in the environment. Interestingly, SOD and Ec-SOD mRNA levels were found to be modulated in an opposite way, the first being upregulated and the latter downregulated. This finding was substantially similar to previous in vitro results that evidenced the induction of the intracellular SOD and the parallel downregulation of Ec-SOD mRNA expression in parasitized oysters [21,22]. In addition, Morga et al. [23] reported that wild oysters injected with *B. ostreae* showed a general downregulation of Ec-SOD expression, probably due to the decrease of phagocytosis. These latter findings would seem to prove that infected oysters display time-related changes in mRNA levels of genes involved in mediating the cellular response to parasite internalization. 

In our study, genes from the HSP family (i.e., HSP70 and HSP90) also showed a consistent response, with a significant mRNA increase in infected oysters. HSP induction observed in our work is likely to be related to oxidative stress generated by the parasitic infections. This finding is in line with previous research showing pathogen-induced expression of heat shock proteins in various host species, including oysters [36]. Overall, these results suggest that HSPs can be used as indicator of pathogenic stress, but not stress resistance, in bivalves. The gene expression of the HSPs in response to stress condition involves the binding of a cytoplasmic protein, the heat shock factor (HSF), to the heat shock element (HSE) in the promoter DNA sequence [37,38,39]. It is noteworthy in this regard that HSF can also activate the promoters of MT genes [40,41], thus contributing to the activation of MT gene families during oxidative stress response.

On the other hand, the observed overexpression of IAP and FAS in infected oysters may indicate that resistance to parasitosis involves modulation of the apoptotic pathway, as previously shown in different invertebrate models [23,42,43,44]. Concomitant upregulation of IAP and FAS seems to indicate that both the parasite and the host rely on modulating apoptosis. Indeed, increased IAP expression is likely due to the parasite’s attempt to manipulate host defense mechanisms and survive within oyster hemocytes. Similarly, the overexpression of GAL can be interpreted as a parasite-induced mechanism for facilitating the internalization process. The relationship between GAL expression and *Bonamia* infection has already been described in *O. edulis* [23,42,45]. 

Overall, the present study first corroborates the occurrence of these parasites along the Italian coast of the central Adriatic Sea, thus confirming that continuous monitoring is critical to establish the health and status of wild oyster populations. Second, our work is a preliminary investigation of the molecular mechanisms expressed by the hemocytes in response to infection with *B. ostreae, B exitiosa* and *M. refringens*, present singularly or in combination. Indeed, qRT-PCR analysis has shown that the presence of parasite DNA may be associated to a general upregulation of host genes related to detoxification and immune response, with the exception of Ec-SOD, whose downregulation might reflect a mechanism for parasite escape before internalization. Such results confirm that the relationships between flat oyster and parasites result in the modulation of common defense mechanisms, ranging from apoptosis to detoxification and oxidative stress protection, which can be reliably predicted by using the proposed gene biomarkers (Figure S1). However, these molecular responses require further studies in order to investigate their association with other aspects of host–parasite interactions, such as the level of infection and the host reproductive cycle.

## 4. Material and methods

### 4.1. Sampling Sites and Oysters

During February 2018, 48 wild juvenile flat oysters (*O. edulis*) (46.6 mm in mean length; 18.1 g in mean weight) were sampled from natural beds widespread on the coast of Civitanova Marche (Province of Macerata, Marche Region, Italy; 3–5 nautical miles offshore). Values of the physicochemical parameters of temperature, salinity, dissolved oxygen and pH measured over the sampling period were 9.5 °C, 35 g L^−1^, 7.5 ppm and 8.1, respectively. 

The oysters were transported to the laboratory of Unità di Ricerca e Didattica di San Benedetto del Tronto (URDIS), University of Camerino in San Benedetto del Tronto (AP, Italy) and tissues of digestive gland and gill were dissected using sterile instruments for every oyster and immediately frozen in liquid nitrogen and stored at −80 °C before the molecular biology analyses. Hemolymph was recovered from the adductor muscle sinus using a 1 mL syringe (needle 0.40 mm × 13 mm), as described by Morga et al. [23]. Animal manipulation was performed according to the recommendations of the local University Ethical Committee and under the supervision of the authorized investigators.

### 4.2. Molecular Detection of Parasites: B. ostreae and M. refringens 

Genomic DNA was extracted from gill and digestive gland tissues using Trizol reagent following the manufacturer’s instructions (ThermoFisher Scientific). For *Bonamia* genus and *M. refringens*, conventional PCR with primers described by the Manual of Diagnostic Tests for Aquatic Animals [46] and Pernas et al. [47] was used. Amplifications were performed in 25 µL volume containing 2 μL genomic DNA template, 12.5 μL of 2X PCR Taq MasterMix (Applied Biological Materials Inc.) and 1 µL of each primer (10 µM; Eurofins MWG Operon). Thermocycling for *Bonamia* genus DNA was for 3 min at 95 °C followed by 40 cycles of 20 s at 95 °C, 30 s at 60 °C and 45 s at 72 °C. Positive samples for the parasites were tested using *Bonamia* species-specific conventional PCR assays [48,49,50]. Thermocycling for *B. ostreae* DNA detection was for 3 min at 95 °C followed by 40 cycles of 30 s at 95 °C, 30 s at 56 °C and 45 s at 72 °C. Thermal cycling conditions for *B. exitiosa* DNA detection were 95 °C for 3 min, followed by 35 cycles of 95 °C for 20 s, 58 °C for 20 s and 72 °C for 45 s. Amplification of *M. refringens* DNA was performed with a nested PCR to increase sensitivity and specificity. Thermocycling was for 3 min at 94 °C followed by 30 cycles of 60 s at 94 °C, 60 s at 55 °C and 60 s at 72 °C. All DNA amplicons were electrophoresed through a 1.0% agarose gel and visualized under a UV light transilluminator by staining with SafeView FireRed (Applied Biological Materials Inc.) (Figure S2).

### 4.3. Quantitative Real-Time PCR (qRT-PCR)

Total RNA was extracted from hemocyte samples using Trizol Reagent following the manufacturer’s instructions (ThermoFisher Scientific). DNase treatment (2U, 25 min, 37 °C; Ambion) was carried out to eliminate genomic DNA contamination. RNA concentration was assessed spectrophotometrically at absorbance of 260/280 nm, and the integrity was confirmed by electrophoresis through 1% agarose gels stained with SafeView Classic (Applied Biological Materials Inc.). The complementary DNA (cDNA) was synthesized from 2 μg of total RNA in 20 μL of total volume reaction using 5X All-In-One RT MasterMix according to manufacturer’s instructions (Applied Biological Materials Inc.). For molecular analyses, a SYBR Green ABI 7300 Real-Time PCR assay was performed with specific primers for SOD, Ec-SOD, MT, GST, HSP70, HSP90, IAP, FAS and GAL target genes (Table 1). The optimized reaction mixture contained 12.5 uL 2X BrightGreen qPCR MasterMix (abm), 1 μL each of forward and reverse primers (10 µM; Eurofins MWG Operon), 1 μL cDNA template and sterile distilled water. Thermo-cycling for reaction was for 10 min at 95 °C, followed by 40 cycles of 15 s at 95 °C and 60 s at 60 °C. Melting curve analysis at 95 °C for 1 min, 55 °C for 30 s and thereafter decreasing fluorescence detection with increasing temperature between 55 and 95 °C demonstrated that a single product was generated for each reaction, confirming the specificity of primer pairs. The efficiency of reactions was determined by performing real-time PCR on serial dilutions of cDNA (Table 1). The expression of the target genes was normalized to glyceraldehyde 3-phosphate-dehydrogenase (GAPDH) and calculated using the method described by Pfaffl [51]. GAPDH has previously been identified as one of the most stable housekeeping genes for similar studies [52]. In addition, absolute quantification was carried out using the standard curve method (Figure S3).

### 4.4. Statistical Analyses

The results were illustrated in scattered dot plots of mRNA expression (1/ΔCT relative to glyceraldehyde 3-phosphate dehydrogenase (GAPDH)) and checked for normal distribution using the Shapiro–Wilk test and homogeneity of variance with Levene’s test; Student’s *t*-test was used to determine statistical differences between infected and non-infected oysters. Differences were considered significant with a probability (P) value less of 0.05. To better understand the interactions between gene biomarkers and parasite infection, principal component analysis (PCA) was performed using XLSTAT Software (version 2015.4.01 Addinsoft SARL).

## Figures and Tables

**Table 1:** List of primers used in this study.

Gene	Forward Sequence (5′–3′)	Reverse Sequence (5′–3′)	References	Efficiency (%)
MT	CTAATTTTACTCCTTCCAAC	CAGGCGACCATTAATTCAC	[53]	99.52
SOD	TCGTCAATGTCAGCGTGAA	AAATGTTGGGGCTGGTGA	[21]	102.87
GST	GGTCGTCAGGGGTCAGTTT	GGTTCCCGTTCTTGAGCA	[21]	102.52
HSP70	AGCAAGCCAGCACAGCA	GCGATGATTTCCACCTTC	[54]	98.27
HSP90	TTTGTGGAACGGGTCAAAA	AACGTCGAGCACAGTCGAG	[21]	95.76
IAP	CTACCTCCCCAGGATTGTCA	CACCACTCTCCTCCATGTCA	[42]	97.56
FAS	TTTGGGCAGTGGTGTAAGTG	TAGCCCTGTTTCTCCACCAG	[42]	98.50
GAL	TCGGAGGTCGCCCTTAAT	TTGCCGTGAACAATCAACA	[22]	99.30
EcSOD	GAGGAGGAAGAGGACCATCC	ATTTTCCTCCGCTTTGTGTG	[42]	97.67
GAPDH	TCCCGCTAGCATTCCTTG	TTGGCGCCTCCTTTCATA	[52]	98.50

## Supplementary Materials

The following are available online at https://www.mdpi.com/2076-0817/9/5/323/s1, Figures S1–S3.
