# Supplementary material for: Transcriptional Alteration of Gene Biomarkers in Hemocytes of Wild Ostrea edulis with Molecular Evidence of Infections with Bonamia spp. and/or Marteilia refringens Parasites
Source: Pathogens. 2020 Apr 26;9(5):323. doi: 10.3390/pathogens9050323 (PMC7281406; doi:10.3390/pathogens9050323)
Supplement: Supplementary file 1 [file pathogens-09-00323-s001.pdf]

Supplementary Materials

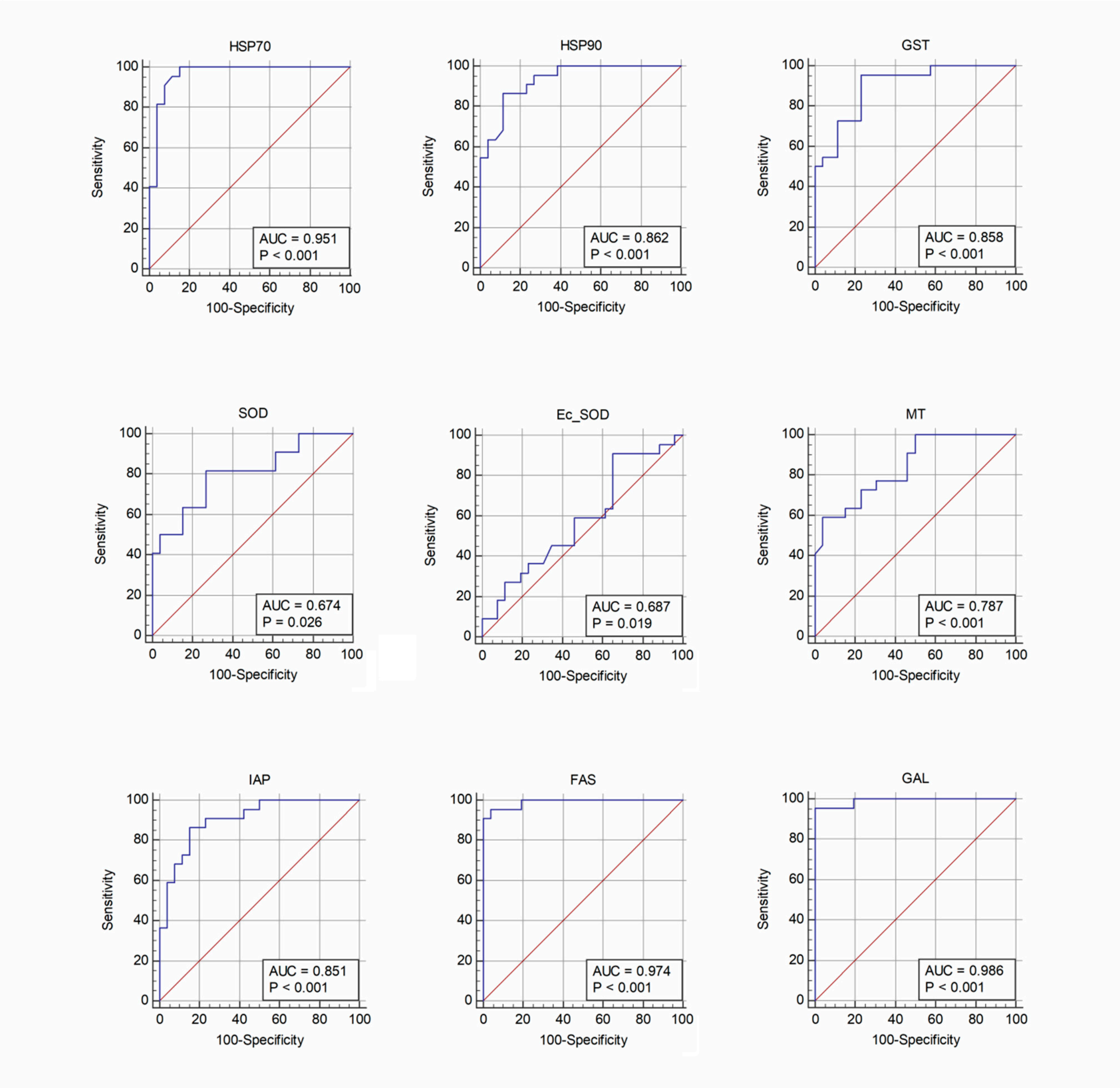

Figure S1. Biomarker potential of target genes in infected oysters. ROC curves generated for heat shock protein - HSP 70 and 90, glutathione S-transferase (GST), superoxide dismutase (SOD), extracellular superoxide dismutase – Ec-SOD, metallothionein (MT), inhibitor of apoptosis - IAP, fas ligand - FAS and galectin - GAL transcription values as markers to distinguish infected from non-infected oysters. Area under curve (AUC), with P value, is given for each gene.

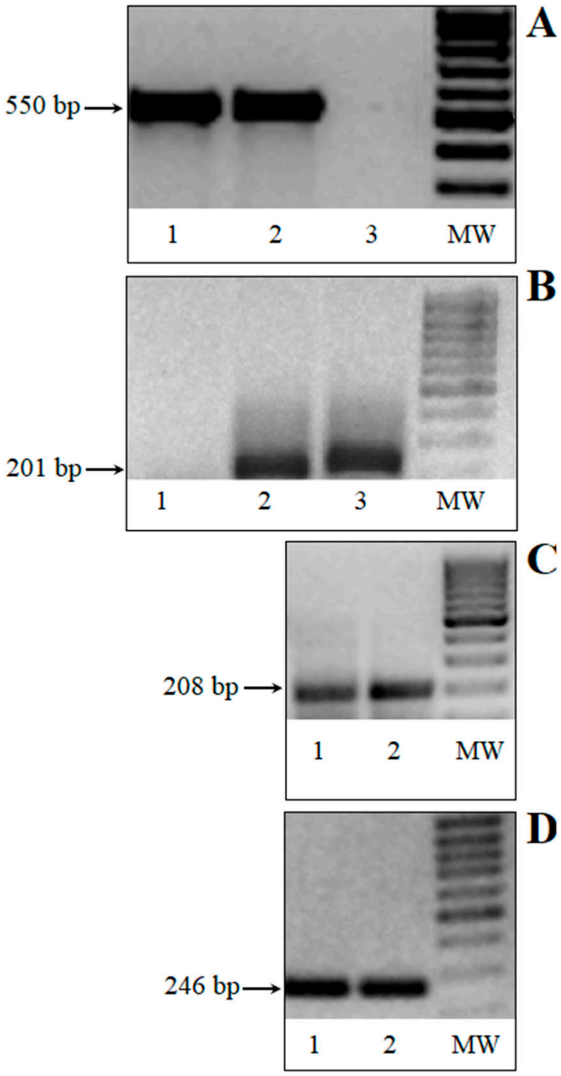

Figure S2. Representative agarose gel electrophoresis after PCR assays performed for A) *Marteilia refringens* (1: gill, 2: digestive gland, 3: negative) B) *Bonamia* spp. (1: negative, 2: gill, 3: digestive gland) C) *Bonamia ostreae* (1: gill, 2: digestive gland) and D) *Bonamia exitiosa* (1: gill, 2: digestive gland). MW: 100 bp molecular marker. Full details are provided in the 'Material and Methods' section of the manuscript.

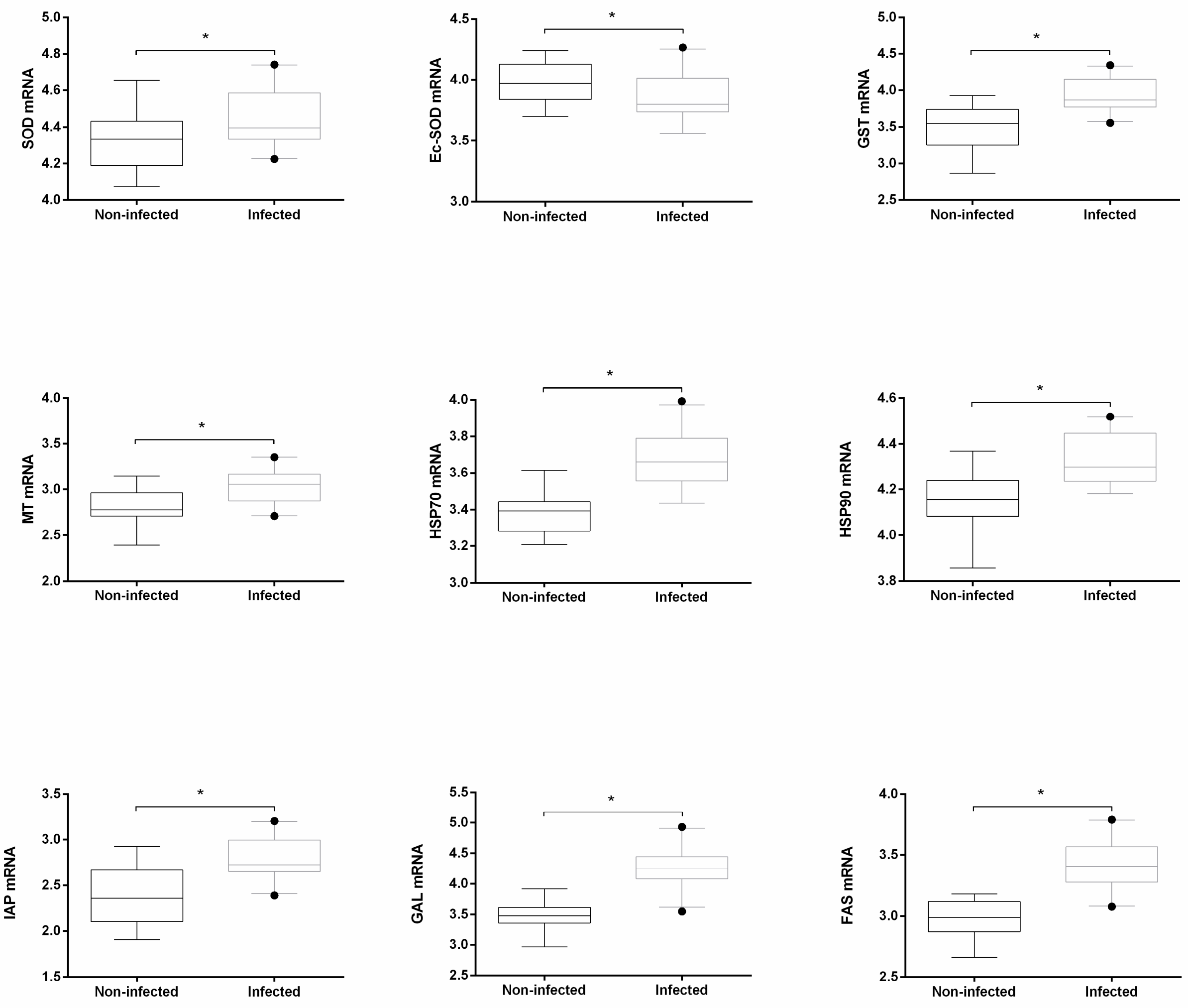

Figure S3. Box-and-whisker plots (medians and 95% confidence intervals) of superoxide dismutase (SOD), extracellular superoxide dismutase (Ec-SOD), glutathione S-transferase (GST), metallothionein (MT), heat shock protein - HSP 70 and 90, inhibitor of apoptosis - IAP, fas ligand - FAS and galectin - GAL transcription values (log copy number) in haemocytes of infected and non-infected groups of oysters *Ostrea edulis*. Obtained cycle threshold (Ct) values were converted into mRNA copy number using standard plots of Ct-value versus copy number. The standard curves were produced using serial dilution of known amounts of the amplicon of interest [1-3]. Statistical significance (*P<0.01) was evaluated by Student's t-test.

References

1. Arukwe, A. Toxicological housekeeping genes: do they really keep the house? *Environmental science & technology* **2006**, *40*, 7944-7949.
2. Cocci, P.; Mosconi, G.; Bracchetti, L.; Nalocca, J.M.; Frapiccini, E.; Marini, M.; Caprioli, G.; Sagratini, G.; Palermo, F.A. Investigating the potential impact of polycyclic aromatic hydrocarbons (PAHs) and polychlorinated biphenyls (PCBs) on gene biomarker expression and global DNA methylation in loggerhead sea turtles (Caretta caretta) from the Adriatic Sea. *The Science of the total environment* **2018**, *619-620*, 49-57, doi:10.1016/j.scitotenv.2017.11.118.
3. Cocci, P.; Capriotti, M.; Mosconi, G.; Palermo, F.A. Transcriptional variations in biomarkers of Mytilus galloprovincialis sampled from Central Adriatic coastal waters (Marche region, Italy). *Biomarkers : biochemical indicators of exposure, response, and susceptibility to chemicals* **2017**, *22*, 537-547, doi:10.1080/1354750X.2017.1315614.
